# Perceived effectiveness and intrusiveness of school security countermeasures among parents, students, and staff

**DOI:** 10.1007/s10669-025-10004-7

**Published:** 2025-02-10

**Authors:** Katie Byrd, Kevin Kapadia, Richard John

**Affiliations:** 1 Department of Psychology, University of Southern California (USC), Los Angeles, CA USA; 2 National Center for Risk and Economic Analysis of Threats and Emergencies (CREATE), University of Southern California (USC), Los Angeles, CA USA

**Keywords:** School security, Safety perceptions, Countermeasures, Educational policy

## Abstract

This study evaluates the perceptions of school security countermeasures among parents, recent students, and staff (*n* = 1105) recruited via Prolific.com. The survey elicited participants’ perceptions of safety and countermeasure effectiveness and invasiveness. Results show broad acceptance of measures like emergency drills, door locks, and security cameras, while more invasive measures, including armed staff, bag inspections, and clear bag policies, generate discomfort. A binary logistic regression found that perceived safety was negatively predicted by a desire for additional countermeasures and positively predicted by perceptions of countermeasures as both comfortable and effective. Socioeconomic factors, like household income, were also positively related to students’ sense of safety. Future research should examine the broader impacts of security measures on student well-being and the school environment.

**Supplementary Information:**

The online version contains supplementary material available at 10.1007/s10669-025-10004-7.

## Introduction

Safety in schools is a major concern for educators, policymakers, and parents. While the purpose of various security measures is to protect students and staff, the effectiveness and perception of these measures can vary significantly across different groups within the school community. Understanding these perceptions is necessary for developing strategies that improve safety and maintain a supportive and non-intrusive educational environment.

Previous studies have shown that while students generally feel safe at school, extensive security measures can sometimes lead to decreased feelings of safety. For example, schools with more countermeasures tend to be associated with lower feelings of safety among students (Mowen & Freng 2019; Schreck & Miller 2003). Additionally, non-white and female students often report feeling less secure than their white and male peers (Fisher et al. 2018; Riehm et al. 2021; Tessler et al. 2024). The socioeconomic status of the neighborhood surrounding a school also plays a significant role in shaping perceptions of safety. When controlling for neighborhood effects, racial differences in safety perceptions diminish, with the perceived safety of the surrounding community emerging as a key predictor of school safety (Bachman et al. 2011; Kitsantas et al. 2004; McCuddy et al. 2024). These findings demonstrate how the relationship between security measures and feelings of safety can vary based on the demographic characteristics of the student population.

Both students and school staff generally support security measures, particularly those that do not affect their privacy or convenience (Booren et al. 2011; Tanner-Smith et al. 2018; Comer et al. 2023). However, countermeasures such as clear bag policies, metal detectors, and video surveillance often receive mixed reviews due to their potential to disrupt daily routines or infringe on privacy (Brown 2005).

There needs to be a greater understanding of staff and parental perspectives on these measures. One recent study found that most Australian teachers felt safe at schools but did not account for countermeasures, nor may it apply to American schools where potential threats differ (Longmuir et al. 2024). While students’ perspectives on school security have been studied, teachers and parents are equally important. Teachers and staff are directly impacted by working in these environments, and parents influence school policies through their roles in school boards, voting for school officials, and deciding where their children attend school. Despite this, there is limited research on how parents and teachers/staff perceive school security measures.

This study aims to evaluate the perceptions of school security countermeasures among parents, recent students, and school staff. By gathering insights from these three groups, the study seeks to understand how different groups perceive the effectiveness and invasiveness of various security measures and how those perceptions impact their feelings of safety and decision to attend/remain at the school.

## Previous research

Previous studies have consistently found that students generally feel safe at school, although more security measures can sometimes correlate with lower feelings of safety (Perumean-Chaney & Sutton 2013; Schreck & Miller 2003; Mowen & Freng 2019). This phenomenon may be explained by Ferraro’s (1995) theory of incivilities, which suggests that highly visible security measures might be perceived as indicators of a high-risk environment, thereby increasing fear among students. Further, the fear of crime at school is influenced by both the conditions of the community and the school, as well as individual student characteristics. Schreck and Miller (2003) found that school security efforts do not predict student fear as effectively as these other factors, and some security measures can even exacerbate fears.

The financial implications of implementing security measures are significant. A study by DeAngelis et al. (2011) revealed that urban school districts spend considerably more on physical security measures than rural districts, often at the expense of mental health services for students. There is a clear need for policies on physical security and students’ psychological well-being.

The presence of law enforcement in schools, such as school resource officers (SROs), has also been a subject of debate. While some studies suggest that SROs can help mitigate problems like bullying and racial tensions (Jennings et al. 2011), others indicate that a higher number of SROs correlates with increased feelings of insecurity among students (Crawford & Burns 2016). Implementing certain security measures can also have significant legal and ethical implications (Hsieh et al. 2023). Beger (2003) highlights the question of Fourth Amendment rights for students due to the increasing presence of police and security protocols in schools. These measures often result in invasive searches and an environment that can detract from the educational experience. These mixed results reaffirm the importance of carefully considering a school’s specific context and needs before implementing security measures.

A recent RAND report (Hollywood et al. 2024) highlighted that education and private buildings are among the most frequently targeted types of soft targets and crowded places (ST-CPs), with attacks often motivated by personal, terrorist, or extremist reasons. It emphasizes the need for a layered security approach, where multiple security measures simultaneously prevent or mitigate attacks. The report also points out that effective prevention measures, such as public awareness of warning signs and threat assessment teams, are critical for stopping attacks before they occur. Additionally, the effectiveness of physical security measures, such as locks and secured entryways, as well as the role of bystanders and security personnel in stopping attacks, is emphasized. These insights are particularly relevant for schools where there is a balance between adequate security and maintaining a conducive learning environment. As schools continue to implement various security measures, understanding how these measures are perceived by different groups—recent students, parents, and staff—is essential.

## Methods

The current study (*n* = 1105) evaluated perceptions of school security countermeasures among three distinct groups: parents, recent students, and staff. Participants were recruited through Prolific.com and provided insights into their experiences with various security measures at their schools. The survey collected data on participants’ perceptions of safety and opinions on different security countermeasures, including their effectiveness, comfort levels, and perceived invasiveness. The USC IRB approved the study as exempt. For a complete version of the survey, please refer to Appendix A.

### Sample

The study included three distinct groups: parents (*N* = 366), recent students (*N* = 366), and staff (*N* = 373). All participants were recruited from Prolific.com, a well-validated online platform for behavioral research (Peer et al. 2023, 2021, 2017; Douglas et al. 2023). Participants were compensated $1.50 upon successful completion of the study. The median completion times for the survey were 4.83 min (25th percentile = 3.95, 75th percentile = 6.25) for staff, 4.71 min (25th percentile = 3.52, 75th percentile = 6.53) for parents, and 4.76 min (25th percentile = 3.83, 75th percentile = 6.20) for recent students. A total of six (1.61%) students, seven (1.88%) parents, and two (0.53%) staff failed the attention check question.

To qualify for the student group, participants had to be over 18 and have attended high school in person within the US in the past four years. For the parent group, participants had to currently be a parent or guardian of a child in K-12 grade. For the staff group, each participant had to currently be a teacher or administrator at a K-12 school (every grade level did not have to be present at the school). Participants who failed the attention check question—seven parents, two staff, and six recent students—were removed from the sample. Demographic information for all three groups, including sex, race, income, and political ideology, is found in Table 1.

**Table 1:** Sex, race, income, and political ideology by group

Sample size (*n*)		Recent students	Staff	Parents
		366	366	373
Gender				
	Female	52.7%	70.0%	46.2%
	Male	44.3%	29.0%	53.0%
	Other	3.0%	1.0%	0.8%
Race/ethnicity				
	Caucasian/White	63.4%	87.9%	82.5%
	Hispanic	18.0%	7.2%	6.3%
	Black or African American	13.9%	4.0%	10.9%
	Asian or Pacific Islander	13.7%	3.2%	4.1%
	Other	1.4%	2.7%	4.9%
Income				
	Less than $10,000	8.4%	1.3%	2.8%
	$10,000–$49,999	32.2%	20.7%	24.9%
	$50,000–$99,999	31.1%	45.2%	39.9%
	$100,000–$149,999	12.8%	23.7%	16.8%
	$150,000 or more	9.2%	8.8%	15.8%
Political ideology				
	1 (Extremely liberal)	20.5%	24.0%	15.3%
	2	23.0%	25.8%	20.8%
	3	16.1%	17.7&	14.3%
	4 (Moderate)	25.6%	16.7%	22.6%
	5	7.7%	7.6%	11.5%
	6	4.9%	5.6%	11.5%
	7 (Extremely conservative)	1.5%	1.5%	3.5%

Percentages may not add to 100% due to rounding

Table 2 summarizes the characteristics of the schools for each group in the study. The recent student group was drawn exclusively from high schools, which are generally larger than elementary and middle schools, while staff and parents represented/were associated with all school types. Approximately, 80% of participants in each group were affiliated with standard public schools, which are public schools that are not specialized, e.g., magnet or charter schools.

**Table 2:** School size and type by group

		Recent students (%)	Staff (%)	Parents (%)
Size of school	1–500 students	14.5	46.6	42.1
	501–1000 students	28.7	34.1	38.0
	1001–1500 students	28.1	8.0	12.8
	More than 1501 students	28.7	10.7	7.1
Type of school	Standard public school	81.4	75.6	81.7
	Magnet or charter school	8.5	9.4	5.5
	Religious/parochial private school	7.1	7.5	6.3
	Non-religious private school	2.5	6.2	2.7
	None of the above	0.5	1.3	3.8

Percentages may not add to 100% due to rounding

### Survey

At the beginning of the survey, participants received specific instructions to ensure consistent responses. Recent students answered questions based on the secondary/high school where they spent the most time. Staff answered questions based on the school where they were currently employed. Parents answered questions for their youngest child in school. All participants were instructed to consider the same school for the entire survey.

Participants first answered questions related to their school, including school size and type. They then responded to questions about their perceptions of safety at school, the impact of safety on their decision to attend the school, and whether they had ever considered switching schools due to safety concerns. Next, participants provided information on the security countermeasures present at their school, identified which countermeasures they would like to see improved or added, and noted any for which they felt uncomfortable. They also indicated how strongly they believed each countermeasure increased their safety and the degree to which certain measures invaded their privacy. Finally, participants answered questions regarding their demographics.

The following eleven countermeasures were identified as the most common in schools (Fisher et al. 2018) and were therefore included for every question involving countermeasures: security cameras, uniformed security, traffic barriers, walk-through metal detectors, bag inspections, clear bag policies, interior door locks, staff and student emergency plans/drills, armed staff, and anonymous reporting systems. In addition, the following additional countermeasures were also included: no firearms policy and threat assessment programs.

## Results

The results are organized as follows: first, countermeasures and the desire for improvements or additions are examined (Sect. 4.1), followed by an analysis of perceptions regarding the effectiveness and comfort with these measures (Sect. 4.2). Concerns related to privacy and overall school safety are then explored (Sects. 4.3 and 4.4), concluding with the impact of security measures on school choice and switching behavior (Sects. 4.5 and 4.6).

### Presence and improvements/additions of countermeasures

**Fig. 1:**
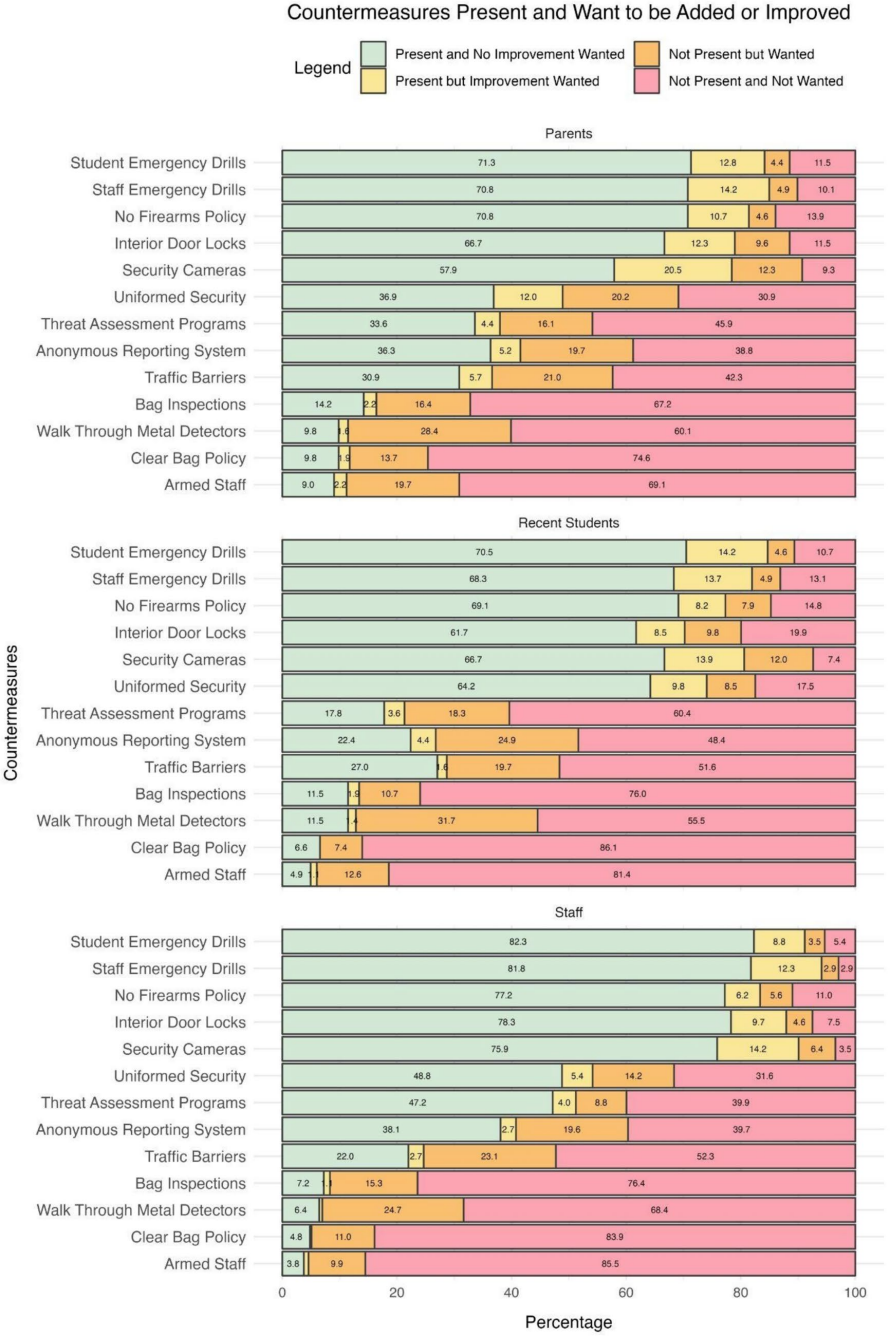
Percentage of participants by group reporting each countermeasure present at school and whether the participant wanted the countermeasure added/improved. Percent labels less than 1% are not included in the figure due to size constraints

Participants were asked to identify the security countermeasures present at their schools and which countermeasures they wanted to add/improve. Figure 1 graphically displays the percentage of each group (staff, parents, recent students) that reported either (1) the countermeasure is present, and no improvement is needed, (2) the countermeasure is present, but they would like it improved, (3) the countermeasure is not present, but they would like it added, or (4) the countermeasure is not present, and they did not select to improve it.

Many countermeasures are currently present at schools, and no improvement was wanted (i.e., green bars in Fig. 1). Across all three groups, the results suggest countermeasures for general threats, specifically emergency drills (staff/student), no firearms policies, interior door locks, and security cameras are widely implemented and not seen as requiring further enhancement by most participants across all groups (> 58%). In addition, students (64%) followed by staff (49%), also reported being satisfied (i.e., not wanting improvement) with the current presence of uniformed security, though this is less frequently noted by parents (37%).

While many countermeasures are present and perceived as sufficient, there are several areas where participants desire improvements to existing countermeasures (i.e., yellow bars in Fig. 1). In particular, security cameras emerged as a top countermeasure for improvements across all groups (14–21%). Both staff and student emergency drills were also highlighted as areas for enhancement across all three groups (9–14%). Interior door locks and uniformed security were also mentioned by parents (12% each) as needing improvement. These results indicate that while many of these countermeasures are widely implemented, participants—especially parents—see room for improvement in surveillance and emergency preparedness areas.

Other countermeasures are not currently present at participants’ schools but were identified as countermeasures that the participants would like to see added (i.e., orange bars in Fig. 1). Among a small portion of all three groups, the most desired but absent countermeasures were walk-through metal detectors (> 25%), traffic barriers (> 20%), and bag inspections (> 11%), indicating a general preference for more stringent entry-point security. Additionally, a portion of all three groups also prioritized anonymous reporting systems (> 19% for all three groups), suggesting a shared concern for improved communication channels. Additionally, parents showed greater support for countermeasures such as armed staff (20%) and uniformed security (20%), compared to the other groups.

Other countermeasures are not currently present at participants’ schools, and participants did not select them as countermeasures they wanted to see added (i.e., red bars in Fig. 1). Across all three groups, armed staff and clear bag policies were the most strongly opposed countermeasures (69–86%), with students and staff showing the highest levels of opposition (> 81%). There was also significant opposition to bag inspections (67–76%) and walk-through metal detectors (56–68%). Additionally, students and staff did not want to add traffic barriers (52%), while students did not want to add threat assessment programs (60%). These results suggest that while participants generally support security enhancements, there is notable resistance among most participants to adding certain countermeasures, especially those related to the introduction of armed staff and strict entry controls.

### Effectiveness and comfortableness with countermeasures

**Fig. 2:**
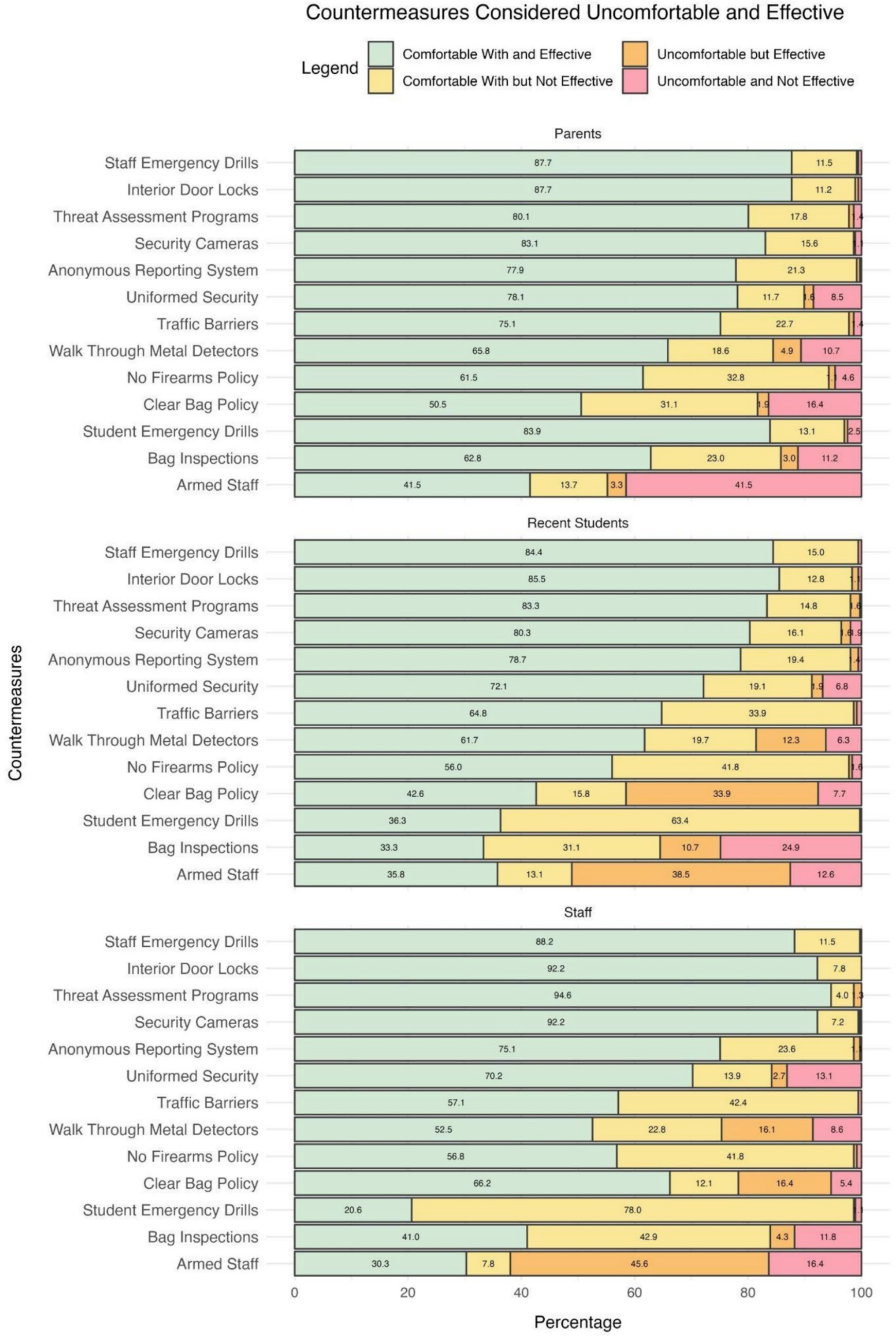
Percentage of participants by group viewing each countermeasure as effective and comfortable. Percent labels less than 1% are not included in the figure due to size constraints

Participants were asked to identify the security countermeasures which they believed to be effective and any countermeasures with which they were uncomfortable. Figure 2 shows the percent of each group (staff, parents, recent students) that reported (1) the countermeasure is effective, and I am comfortable with it; (2) the countermeasure is not effective, but I am comfortable with it; (3) the countermeasure is effective, but I am not comfortable with it, or (4) the countermeasure is not effective, and I am not comfortable with it.

Several countermeasures were widely considered comfortable and effective among all three groups (i.e., green bars in Fig. 2). The highest-rated measures included staff emergency drills, interior door locks, security cameras, and threat assessment programs (> 80% across all three groups). In addition, anonymous reporting systems and uniformed security were viewed positively (> 70% across all three groups). Interestingly, 84% of parents viewed student emergency drills as comfortable and effective, compared to only 36% of recent students and 21% of staff.

Several countermeasures were considered comfortable but ineffective across all groups (i.e., yellow bars in Fig. 2). The no firearms policy showed high levels of comfort but perceived ineffectiveness, with all three groups expressing concerns (33–42%). Bag inspections and traffic barriers were another area where all three groups felt comfortable but did not believe the measure was effective (23–43%). Anonymous reporting systems were viewed as comfortable but ineffective by a portion of all three groups (19–24%). Some differences between the groups emerged. For example, student emergency drills were seen as ineffective despite being comfortable by both staff (78%) and students (63%), compared to parents (13%). Alternatively, clear bag policies were viewed as comfortable but ineffective by parents (31%), compared to students (16%) and staff (12%). These results suggest that, while participants generally feel comfortable with these measures, there is notable skepticism about their effectiveness, particularly student emergency drills, no firearms policies, and bag inspections, with the highest skepticism by staff and students.

Overall, staff and recent students reported significantly more discomfort with certain countermeasures than parents, though they still acknowledged the effectiveness of these measures (i.e., orange bars in Fig. 2). Armed staff was the most frequently identified uncomfortable but effective measure across all groups, with staff reporting the highest amount (46%), followed by students (39%) and parents (3%). Clear bag policies were another countermeasure that was seen as effective but uncomfortable, particularly for students (34%) and staff (15%), with a smaller percentage of parents (2%). Walk-through metal detectors and bag inspections were also considered uncomfortable but effective by staff, students, and parents (3–16%).

Several countermeasures were identified as both uncomfortable and ineffective across all groups (i.e., red bars in Fig. 2). Armed staff stood out as the most frequently cited uncomfortable and ineffective measure, specifically among parents (42%), followed by staff (16%), then students (13%). Bag inspections were also considered both uncomfortable and ineffective by all three groups (11–25%). The clear bag policy and walk-through metal detectors were seen as uncomfortable and ineffective by parents (16 and 11%, respectively), while uniformed security was cited as uncomfortable and ineffective by staff (13%). Overall, these results indicate that the majority (> 59%) of participants across all three groups are comfortable with all of the countermeasures (i.e., green plus yellow bars in Fig. 2), except for armed staff. Additionally, the majority of participants (> 50%) across all three groups think all of the countermeasures are effective (i.e., green plus orange bars in Fig. 2), except for bag inspections and student emergency drills for staff and students and armed staff for parents.

### Invasion of privacy and school safety

While some measures may effectively mitigate physical threats, their presence can signal a dangerous environment or an invasion of privacy, reducing the overall feeling of safety and comfort, especially for students and staff who spend the most time within the school. Participants in the study rated four specific security measures—bag inspections, clear bag policies, security cameras, and walk-through metal detectors—on the extent to which they were perceived as invasions of privacy.

**Fig. 3:**
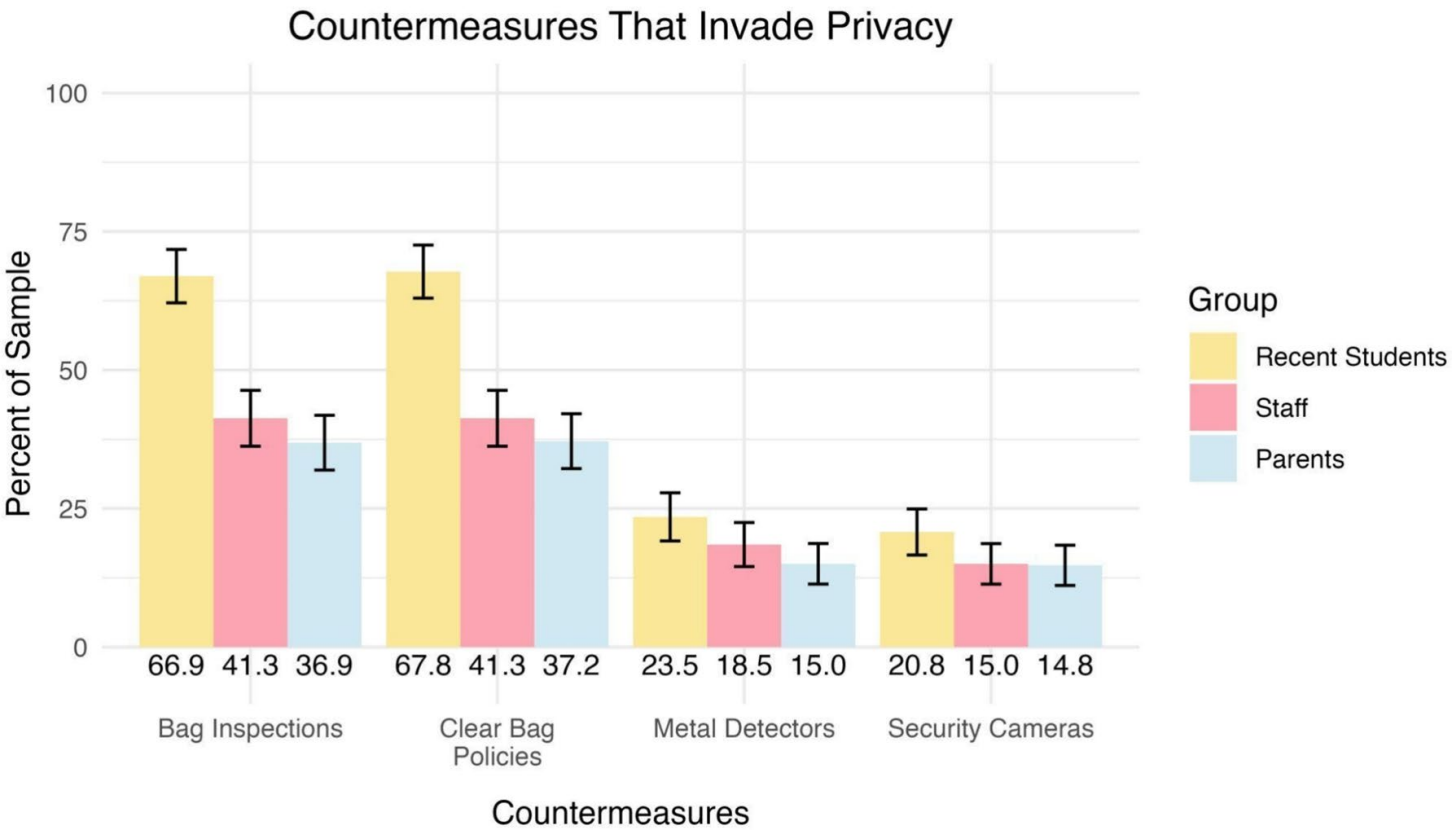
Percentage of participants by group who felt each of four countermeasures would invade their privacy. Group percentages are included at the bottom of each column. Error bars represent 95% confidence intervals

Figure 3 presents the percentage of participants from each group who indicated that these countermeasures invaded their privacy. Across all three groups, bag inspections and clear bag policies were consistently viewed as the most invasive. In contrast, security cameras and walk-through metal detectors were generally perceived as less intrusive. Notably, students were significantly more likely than parents and staff to rate bag inspections and clear bag policies as invasive. Approximately, two-thirds of recent students identified these measures as privacy invasions. Conversely, fewer than a quarter of respondents from any group identified metal detectors and security cameras as privacy-invasive.

### Impact of security countermeasures on school safety

**Fig. 4:**
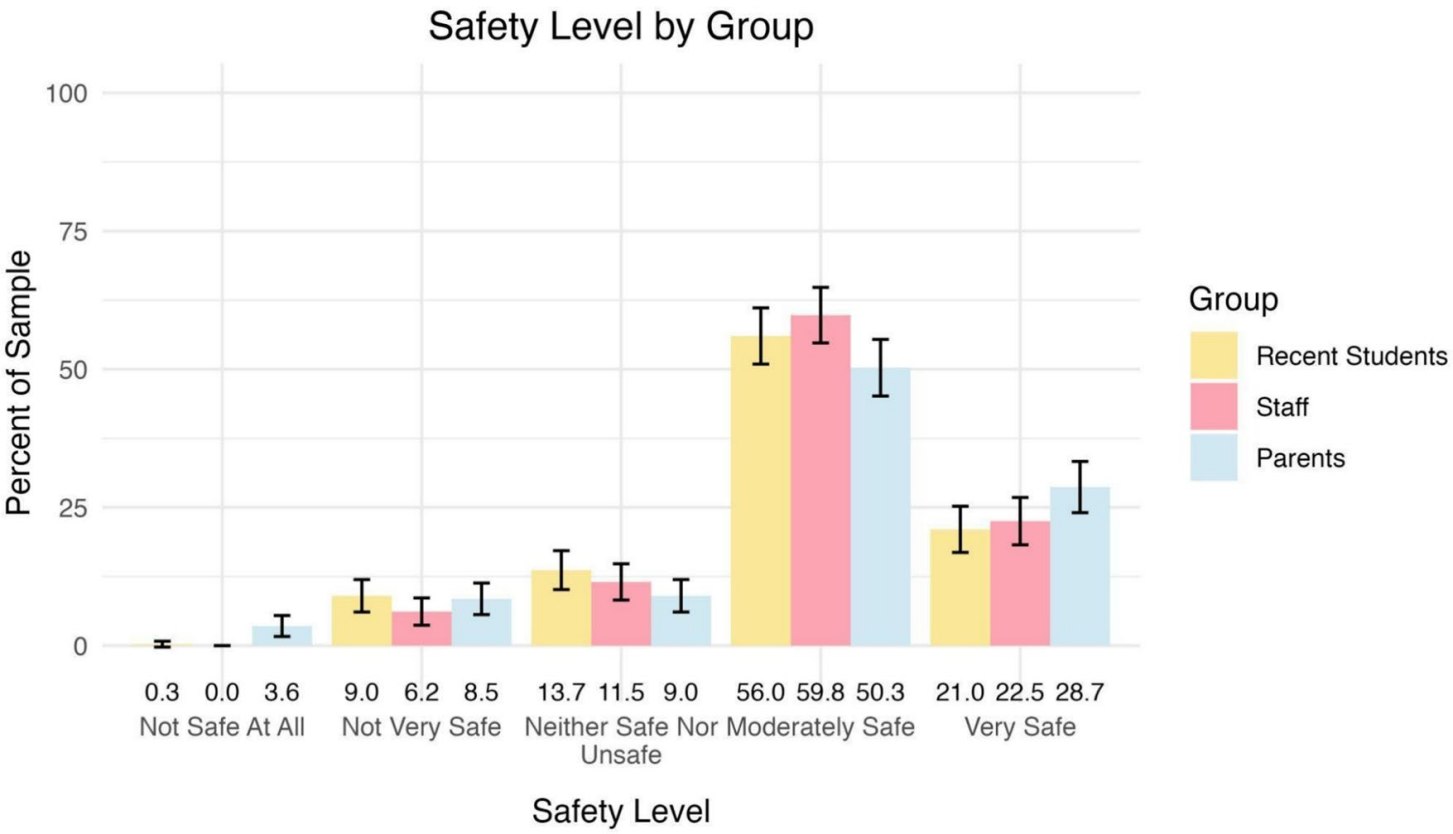
Percentages of participants by group perceiving various levels of school safety. Group percentages are included at the bottom of each column. Error bars represent 95% confidence intervals

Participants reported their overall sense of safety at school. As shown in Fig. 4, the majority of participants (> 75%) indicated feeling moderately safe or very safe, while fewer than 10% reported feeling unsafe (i.e., selecting “Not safe at all” or “Not very safe”). Overall, responses were consistent across the three groups. Parents reported slightly higher percentages for both “Very safe” and “Not safe at all,” while recent students and staff reported marginally higher percentages for “Neither safe nor unsafe” and “Moderately safe.”

A binary logistic regression (BLR) was conducted using the *lm* function in R to predict participants’ sense of safety based on various predictors. Safety responses were aggregated to create a dichotomous variable as follows: “Not safe at all,” “Not very safe,” and “Neither safe nor unsafe” were coded as 0, while “Moderately safe” and “Very safe” were coded as 1. The decision to group “Neither safe nor unsafe” with responses coded as 0 was based on its lack of a definitive positive sense of safety, reflecting our research focus on identifying predictors of a clear sense of safety. However, we acknowledge that this classification simplifies the neutral position “Neither safe nor unsafe.” We chose this approach to streamline the analysis but recognize the need to carefully interpret results considering this limitation.

The predictors in the regression included (1) Satisfied—Participants’ satisfaction with the current countermeasures at their school, measured by the number of countermeasures present at their school that they did not want to be improved (*M* = 5.39, SD 2.65), (2) Unwanted—The number of countermeasures not present at their school that they did not want to add (*M* = 6.04, SD 2.18), (3) Add—The number of countermeasures not present at their school that they want to add, (4) Comfortable + Effective—The number of countermeasures participants found both comfortable and effective (*M* = 8.98, SD 3.23), (5) Uncomfortable + Effective—The number of countermeasures they found uncomfortable but effective (*M* = 0.83, SD 1.10), and (6) Uncomfortable + Ineffective—The number of countermeasures they found uncomfortable and ineffective (*M* = 3.36, SD 2.72). The regression models also included predictors related to participant’s school and demographic information.

The results, presented in Table 3, reveal that several factors significantly influenced participants’ perceived sense of safety. The correlation between the predictors for each logistic regression can be found in Appendix B. The number of countermeasures participants wanted to be added to their school was negatively associated with perceived safety across all three groups (students: OR 0.59, CI 0.42–0.80, parents: OR 0.69, CI 0.57–0.82, staff: OR 0.50, CI 0.33–0.72). Conversely, the number of countermeasures participants found both comfortable and effective was positively associated with perceived safety among students (OR 1.15, CI 1.03–1.30) and parents (OR 1.13, CI 1.01–1.27). Additionally, higher household income was linked to a greater sense of safety among students (OR 1.28, CI 1.08–1.54). While the effect sizes (*R*^2^) in the logistic regression analysis were relatively low, the primary goal of the binary logistic regression was to detect relationships between predictors and the outcome variable rather than to use the model for predictive purposes. These findings suggest that participants’ sense of safety is shaped by the presence or absence of security measures, their perceptions of the comfort and effectiveness of those measures, and certain demographic factors.

**Table 3:** Binary logistic regressions by group predicting how safe participants feel at school

Predictors	Students			Parents			Staff		
	Odds ratios	CI	*p*-Value	Odds ratios	CI	*p*-Value	Odds ratios	CI	*p*-Value
Intercept	2.05	0.06–76.12	0.689	1.36	0.20- 9.56	0.755	9.60	0.17- 623.7	0.277
Satisfied	1.03	0.82–1.27	0.792	0.93	0.81- 1.07	0.302	1.02	0.79- 1.29	0.891
Unwanted	0.85	0.67- 1.07	0.177	0.98	0.86- 1.12	0.773	0.81	0.60- 1.08	0.148
Add	0.59	0.42- 0.80	**0.001**	0.69	0.57- 0.82	** < 0.001**	0.50	0.33- 0.72	** < 0.001**
Comfortable + effective	1.15	1.03- 1.30	**0.016**	1.13	1.01- 1.27	**0.031**	1.05	0.92- 1.19	0.501
Uncomfortable + ineffective	1.17	0.82- 1.72	0.394	1.01	0.81- 1.27	0.929	1.20	0.81- 1.85	0.383
Uncomfortable + effective	1.14	0.87- 1.53	0.357	1.13	0.81- 1.87	0.533	1.21	0.87- 1.72	0.259
School type	1.62	0.71- 4.04	0.274	0.97	0.42- 2.44	0.945	1.03	0.50- 2.23	0.933
Sex	0.65	0.33- 1.28	0.219	1.01	0.55- 1.88	0.968	1.83	0.94- 3.54	0.073
Race	1.11	0.60- 2.10	0.749	1.03	0.49- 2.26	0.943	1.52	0.55- 5.11	0.455
Income	1.28	1.08- 1.54	**0.005**	1.22	0.99- 1.51	0.057	1.08	0.88- 1.34	0.463
Political ideology	0.99	0.80- 1.22	0.892	1.18	0.99- 1.42	0.065	1.05	0.86- 1.29	0.659
Education	2.03	0.59- 6.47	0.240	1.34	0.68- 2.62	0.399	1.40	0.46- 3.88	0.536
*R*^2^ Tjur	20.9%			13.5%			18.3%		

Significant values, *p* < 0.05 are bolded. Satisfied is the number of countermeasures present and participants indicated they did not want to improve. Unwanted is the number of countermeasures not present and participants indicated they did not want added. Add is the number of countermeasures not present but participants indicated they wanted to add. Comfortable + Effective is the number of countermeasures participants did not indicate made them uncomfortable and found effective. Uncomfortable + Effective is the number of countermeasures participants indicated made them uncomfortable but found effective. Uncomfortable + ineffective is the number of countermeasures participants indicated made them uncomfortable and found ineffective. The fourth options—Improvement and Comfortable + Ineffective—were not included in the regression since they are linearly dependent (100%—the sum of the other three categories). Due to sample size constraints, Religious/Parochial Private School, Non-Religious Private School, and Magnet/Charter schools were combined. Private/Charter School is coded: Public = 0, Private/Charter = 1. Sex is coded: Male = 0, Female = 1, with “Other” responses removed from the analysis due to small sample sizes. Race is coded: White/Caucasian = 0, Non-White/Caucasian = 1. Education was coded: Less than a bachelor’s degree = 0, A bachelor’s degree or higher = 1. The income variables were coded Less than $10,000” = 0, $10,000–$19,999 = 0, $20,000–$29,999 = 1, $30,000–$39,999 = 1,$40,000–$49,999 = 2, $50,000–$59,999 = 2, $60,000–$69,999 = 3, $70,000–$79,999 = 3, $80,000–$89,999 = 4, $90,000–$99,999 = 4, $100,000 or more = 5. Political Ideology is a self-reported measure from 1 to 7 where 1 = Extremely liberal and 7 = Extremely conservative

### Impact of security countermeasures on school decision

**Fig. 5:**
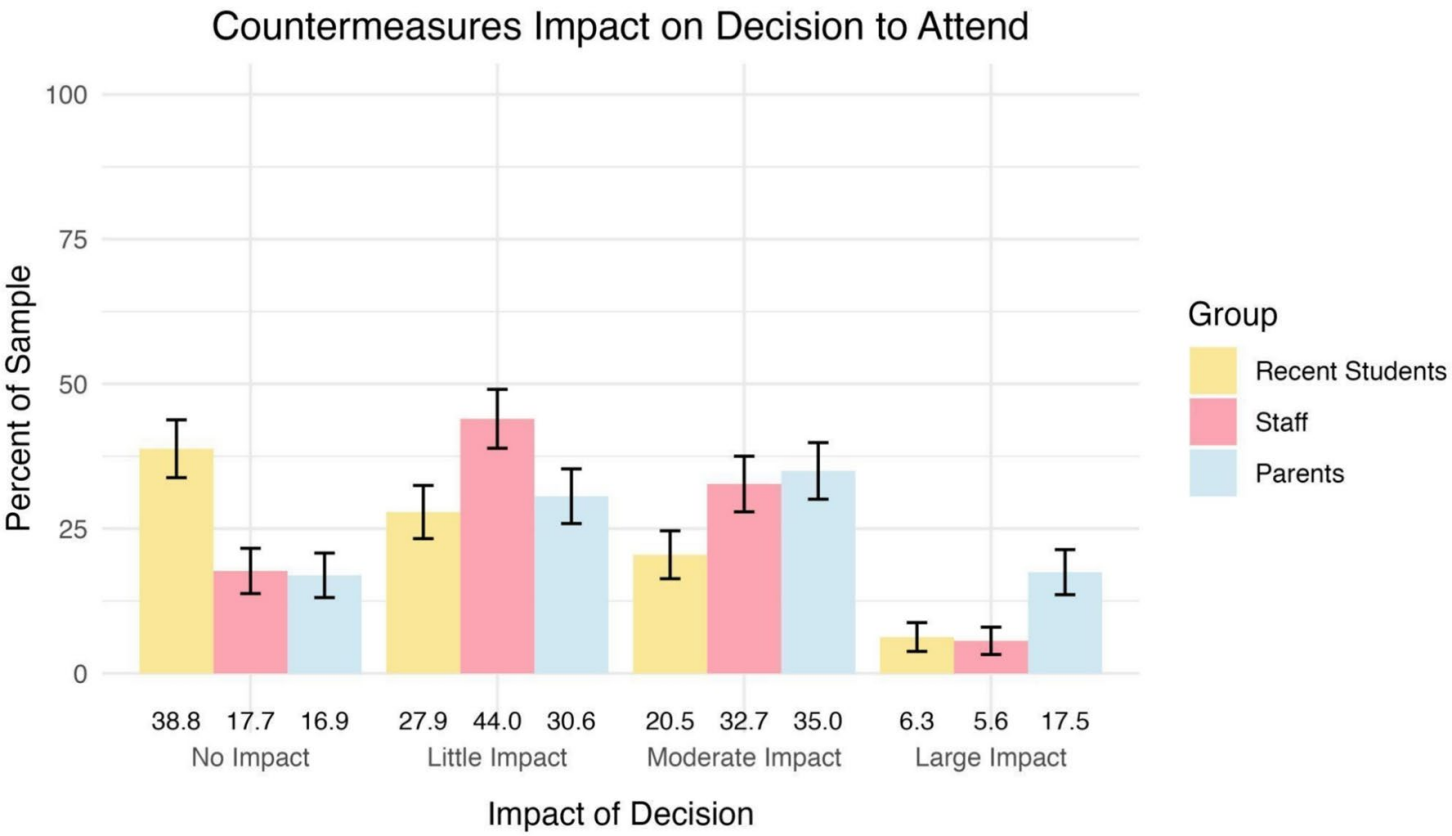
Percentages of participants by group indicating various levels of impact of countermeasures when choosing whether to attend or remain at a school. Error bars represent 95% confidence intervals

The influence of security countermeasures on participants’ decisions to attend or remain at a school was also evaluated. Figure 5 shows that while many participants indicated that security measures had little to no impact on their decision, a notable proportion reported a moderate to large impact, particularly among staff and parents.

### Impact of security countermeasures on school switching

**Fig. 6:**
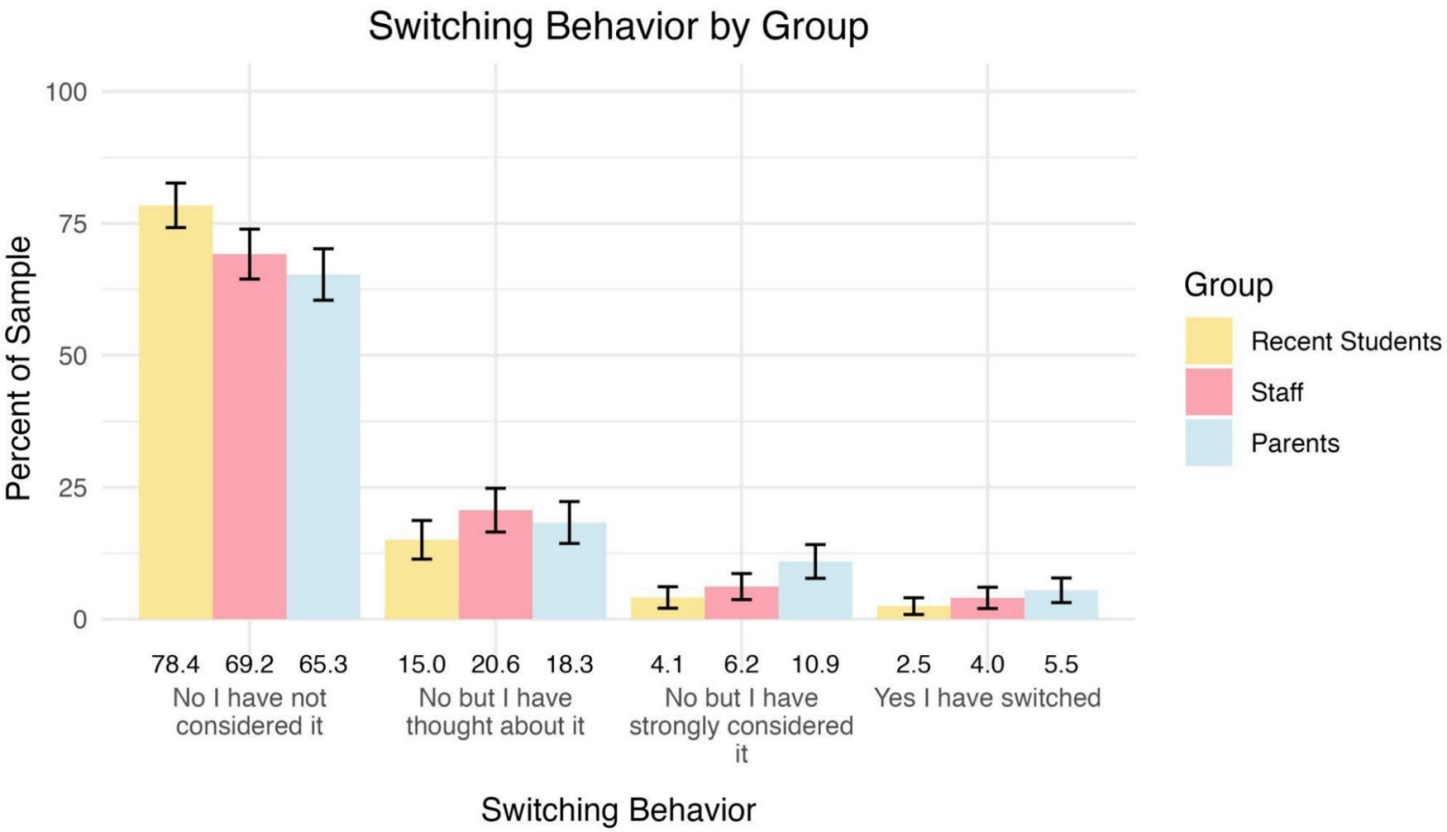
Percentages of participants by group who considered switching schools. Error bars represent 95% confidence intervals

Participants were asked if they had considered switching schools due to safety concerns. Figure 6 demonstrates that the majority have not considered switching schools. However, a small percentage had switched schools, while a slightly larger portion had at least thought about it.

## Discussion

This study evaluated the perceptions of school security countermeasures among parents, students, and staff. The findings provide important insights into how these different groups perceive the effectiveness and privacy implications of various security measures implemented in schools. Across all three groups, there was broad satisfaction with implementing several general security measures, including staff/student emergency drills, interior door locks, no firearms policies, and security cameras. Most participants from each group indicated that these measures were present and did not require improvement. These results suggest a widespread perception that these countermeasures effectively contribute to a safe school environment without causing significant concern or opposition.

The high levels of comfort and effectiveness associated with these measures suggest that these forms of security have been normalized and accepted in school environments. Participants likely view them as essential without being overly intrusive. For instance, security cameras, emergency drills, and no firearms policies are often passive, allowing for surveillance and preparedness without creating an uncomfortable atmosphere. This balance between visibility and subtlety may explain why these measures are widely accepted.

Interestingly, uniform security was one measure that saw slightly more variance in satisfaction across the groups. While 64% of students and 49% of staff reported no need for improvement, only 37% of parents felt similarly. This finding could be attributed to parents’ desire for more visible countermeasures. In contrast, students and staff may be more concerned with maintaining a non-disruptive environment where security personnel are less visible.

While many countermeasures were accepted, there was apparent discomfort with more invasive security measures. The findings show that many participants—especially students and staff—felt uncomfortable with and did not want to add several countermeasures, specifically armed staff, clear bag policies, bag inspections, and walk-through metal detectors. Armed staff was a particularly divisive measure, with a substantial percentage of participants expressing discomfort and no desire to add the countermeasure across all groups. While some participants acknowledged the potential effectiveness of armed staff in preventing or mitigating threats, the negatives of their presence appear to outweigh the perceived benefits for many. Bag inspections and clear bag policies were similarly rated as uncomfortable by a significant percentage of students and staff, who reported feeling that these measures were both invasive and not wanted.

These findings reflect a growing concern that while stringent security protocols might prevent certain threats, they can also affect the sense of trust and safety schools aim to establish.

While most participants viewed walk-through metal detectors as comfortable and effective, most still reported that they were not present at their school and did not want to add them. Regarding privacy invasion, students were particularly sensitive to these measures, especially bag inspections and clear bag policies, compared to parents and staff. This heightened sensitivity could be because students view these measures as violating personal space and autonomy. On the other hand, parents likely see these inconveniences as necessary trade-offs for enhanced safety, resulting in less opposition.

While basic security measures were generally accepted, the study highlighted some key differences in the desire for improvements or additional measures. Parents, in particular, showed a slightly higher interest in enhancing countermeasures. This finding suggests that parents, who are often more removed from the day-to-day school environment, may feel a heightened sense of threat, pushing them to advocate for improvements even to measures that students and staff perceive as sufficient.

The tension between parents’ desire for increased visible security and students’ and staff’s discomfort with such measures points to a greater issue: the need to balance the psychological impacts of security measures with their physical effectiveness. Students and staff who experience school life daily may be more likely to feel that measures like armed staff, clear bag policies, and bag inspections disrupt the educational space and contribute to a sense of surveillance rather than safety.

The discomfort expressed by participants, particularly toward armed faculty/staff, highlights a significant challenge in implementing certain security measures. Arming staff would require cooperation and coordination with staff members, yet as a group, they showed the highest level of discomfort with this measure. This discomfort could be attributed to concerns about the potential escalation of violence or their comfort levels and/or experience with handling a weapon.

The logistic regression provided insight into how different factors influence perceived safety at school. One key finding was the negative association between the number of countermeasures participants wanted added to their school and their perceived sense of safety across all groups (students, parents, and staff). This result suggests that participants who feel less safe may advocate for more security measures, indicating a potential lack of confidence in the current level of security. Conversely, participants who felt safe may not see the need for additional measures, which could explain why they reported fewer desired security enhancements.

On the other hand, the number of countermeasures that participants found both comfortable and effective was positively associated with perceived safety among students and parents. This finding highlights the importance of security measures being perceived as non-intrusive and effective in contributing to a sense of safety. When students and parents feel that more security measures are effective and appropriate, they are more likely to perceive their environment as safe. The absence of this association for staff suggests that other factors, such as concerns over the potential day-to-day impact of these measures on the learning environment, may influence their views on comfort and effectiveness.

Household income was positively associated with students’ perceived sense of safety. This result suggests that students from higher-income households may feel safer due to increased access to resources, greater exposure to schools with more robust security infrastructure, or a general sense of privilege that translates into feeling more secure. This finding aligns with existing literature showing that socioeconomic status can influence how individuals perceive risks and threats, with those from higher-income households often reporting lower levels of perceived vulnerability.

This study provides evidence that perceptions of safety are not just about the presence of countermeasures but are shaped by how these measures are perceived through experience, personal comfort levels, and broader socioeconomic factors. Policymakers and school administrators should consider these aspects before adding additional countermeasures and when evaluating the effectiveness of current security protocols. Measures such as armed staff or clear bag policies that seem objectively effective could still generate discomfort or resistance, particularly among students and staff most directly affected by them. Further, compared to parents’ relatively greater desire for increased security, the contrast between student and staff discomfort with invasive measures like armed staff or bag inspections indicates the need for further discussion. Schools may benefit from engaging with all stakeholders—students, parents, and staff—when developing or refining security protocols.

This study provides practical recommendations to school administrators considering adding or enhancing countermeasures. For each of the three stakeholder groups, we have characterized common countermeasure alternatives in terms of feelings of comfort and perceptions of effectiveness. Students, staff, and parents would be expected to welcome additional or enhanced deployment of countermeasures perceived as effective and not creating discomfort. Fortunately, most of the countermeasures we studied are viewed as both effective and comfortable. Countermeasures that are comfortable but perceived as ineffective, e.g., student emergency drills, would be expected to be neither welcomed nor opposed. Educating students, staff, and parents about why and how the countermeasures are effective would be expected to create greater acceptance. For countermeasures perceived as effective but uncomfortable, e.g., armed staff, there may be ways to mitigate sources of discomfort. Fortunately, countermeasures are rarely viewed as both uncomfortable and ineffective. School administrators would be well advised to avoid such countermeasures, e.g., parents’ view of armed staff, unless perceptions of effectiveness or discomfort can be changed.

## Limitations & future research

While this study provides valuable insights into perceptions of school security countermeasures among parents, recent students, and staff, several limitations should be considered. First, the participants were recruited from Prolific.com, which, despite being a well-validated platform for behavioral research, may not fully represent the broader population of parents, recent students, and staff across various geographic regions and school types, limiting the generalizability of the findings. Next, although the study included a comprehensive list of common security countermeasures, there may be additional measures that were not included that are relevant to some schools. Additionally, the study did not assess these measures’ effectiveness in reducing violence or crime incidents.

Future research could address these limitations and explore the topic in several ways. Obtaining a representative stratified sample to ensure it includes participants from different regions and socioeconomic backgrounds would enhance the generalizability of the findings and provide a more comprehensive understanding of the issue. Investigating the psychological and behavioral impact of security measures on recent students, parents, and staff could provide crucial information on how these measures affect the school environment and student well-being.

Further research could also explore the policy implications of these findings, providing recommendations for school administrators and policymakers on implementing security measures that balance safety, privacy, and comfort for all school stakeholders. By addressing these areas, future research can build on the current study’s findings and contribute to a more comprehensive understanding of school security measures and their impact on school communities.

## Conclusion

This study provides insights into the perceptions of school security countermeasures among parents, students, and staff. Overall, the findings demonstrate that while basic security measures such as emergency drills, door locks, and security cameras are widely accepted and viewed as effective, more invasive measures—such as armed staff, bag inspections, and clear bag policies—generate discomfort, particularly among students and staff. The results also reveal a tension between parents’ desire for increased security measures and students’ and staff’s preference for less intrusive options, reflecting different priorities and experiences between groups. The logistic regression analysis highlighted that participants’ sense of safety is influenced by the presence of security measures and their perceptions of the comfort and effectiveness of countermeasures in general. Additionally, socioeconomic factors, such as household income, play a role in students’ sense of security, indicating that feelings of safety can come from both individual and contextual factors. These findings highlight the importance of school administrators and policymakers balancing security protocols with the need to consider comfort, privacy, and the psychological well-being of students and staff. Involving all groups in conversations about security measures could result in more effective and widely accepted safety strategies.

## Supplementary Information

Below is the link to the electronic supplementary material.

Supplementary file1 (PDF 272 KB)

## Data Availability

For data requests, please contact Richard John at richardj@usc.edu.
