# Supplementary material for: Perceived effectiveness and intrusiveness of school security countermeasures among parents, students, and staff
Source: Environ Syst Decis. 2025 Feb 10;45(1):11. doi: 10.1007/s10669-025-10004-7 (PMC11811474; doi:10.1007/s10669-025-10004-7)
Supplement: Supplementary file 1 — Supplementary file1 (PDF 272 KB) [file 10669_2025_10004_MOESM1_ESM.pdf]

Appendix A

University of Southern California Department of Psychology and CREATE
INFORMATION/FACTS SHEET FOR EXEMPT NON-MEDICAL RESEARCH
Perceptions of Security Measures at Schools

You are invited to participate in a research study about security measures at schools in the United States. This document provides information about this study. You must be 18 or older to participate, and your participation is voluntary. Please take as much time as you need to read this information sheet, and feel free to print it for your records.

You will be asked to provide information about your experiences and views of security measures. You will also be asked to indicate your sex, age, ethnicity, and income as background factors. This survey is anticipated to take no more than 5 minutes to complete. However, we expect that most people will finish it more quickly. You will be compensated $1.50 for your time.

There are no anticipated risks to your participation in this study. You can take as many breaks as needed, as well as discontinue your participation at any time for any reason. There will be no information obtained in connection with this survey that can identify you. Your name, address or other information that may identify you will not be linked to your responses. Only the members of the research team, the funding agency (the U.S. Department of Homeland Security) and the University of Southern California's Human Subjects Protection Program (HSPP) may access the data. The HSPP reviews and monitors research studies to protect the rights and welfare of research subjects. The data will be stored indefinitely on password protected researcher computers in a locked room. In addition, the data files themselves also will be password protected. The anonymous data may be used for future research. If you do not want your data used in future studies, you should not participate.

This study is funded by the Department of Homeland Security (DHS) via Science and Technology (S&T) Office of University Programs (OUP) COE SENTRY.

Investigator Contact Information: If you have any comments, concerns, or questions regarding the conduct of this research please contact Richard John via email at richardj@usc.edu, Katie Byrd via email at ksippel@usc.edu, or Kevin Kapadia at kevinkap@usc.edu.

IRB Contact Information: If you have questions, concerns, or complaints about your rights as a research participant or the research in general and are unable to reach the research team, or if you want to talk to someone independent of the research team, please contact the University Park Institutional Review Board (UPIRB), Credit Union Building (CUB), Third Floor #310, Los Angeles, CA 90089-0702; via phone at (213) 821-5272, Fax at (213) 821-5276 or e-mail at upirb@usc.edu.

If you consent, click to continue

Page Break

Are you currently a teacher or administrator at a K-12 school? (Every grade level does not have to be present at the school)

- Yes
- No

Page Break

What is your Prolific ID? (Please note that this response should autofill with the correct ID)

____

We are interested in what you think about security measures used for K-12 grade schools in the United States. You will be asked about your experiences with and beliefs about such measures. For the following questions please answer based on your personal experiences with the school you work at. If you have worked at multiple schools please choose the school that you are currently working at. You will use the same school for the entire survey.

What type of school do you work at?

- Standard Public School (not including magnet and charter schools)
- Magnet or Charter School
- Religious/Parochial Private School
- Non-Religious Private School
- None of the above

How large is your school?

- 1-500 students
- 501-1000 students
- 1001-1500 students
- More than 1501 students

What grade levels are present at your school? (Select all that apply)   Please note that the answer choices provided may not precisely match the grade levels present at your specific school. In such cases, please select the closest option that aligns with the grade levels available in your school.

- Elementary School (K-5)
- Middle School (6-8)
- High School (9-12)

Page Break

How safe do you feel at your school?

- Not safe at all
- Not very safe
- Neither safe nor unsafe
- Moderately safe
- Very safe

How do the security countermeasures that your school has impact your decision to work there?

- ◯ No impact
- ◯ Little impact
- ◯ Moderate impact
- ◯ Large impact

Have you ever switched to another school due to safety concerns?

- ◯ Yes I have switched
- ◯ No but I have strongly considered it
- ◯ No but I have thought about it
- ◯ No I have not considered it

Page Break

Which of the following security countermeasures does your school have? (Select all that apply)

	Confident the school has this countermeasure	Unsure if the school has this countermeasure	Confident the school does NOT have this countermeasure
Security cameras	○	○	○
Uniformed security or law enforcement officers	○	○	○
Plain clothes (undercover) security or law enforcement officers	○	○	○
Barriers to prevent vehicles from plowing into buildings or crowds	○	○	○
Walk-through metal detectors	○	○	○
No firearms policy	○	○	○
Bag inspection	○	○	○
Clear bag policy	○	○	○
Threat assessment programs	○	○	○
Interior door locks	○	○	○
Faculty/Staff emergency plan trainings/drills	○	○	○
Student emergency plan trainings/drills	○	○	○
Armed faculty/staff	○	○	○
Anonymous suspicious activity reporting system	○	○	○

Bulletproof glass	○	○	○
ID checks/badges (for students and teachers)	○	○	○
ID checks/badges (for visitors)	○	○	○
Metal entrance turnstiles	○	○	○

Page Break

Which of the following security countermeasures would you like to **add/improve** at your school? (Select all that apply)

- ☐ Security Camera
- ☐ Uniformed security or law enforcement officers
- ☐ Plain clothes (undercover) security or law enforcement officers
- ☐ Barriers to mitigate vehicles from plowing into crowds
- ☐ Walk-through metal detectors
- ☐ No firearms policy
- ☐ Bag inspection
- ☐ Clear bag policy
- ☐ Threat assessment programs
- ☐ Interior door locks
- ☐ Faculty/Staff emergency plan trainings/drills
- ☐ Student emergency plan trainings/drills
- ☐ Armed faculty/staff
- ☐ Anonymous suspicious activity reporting system
- ☐ Bulletproof glass
- ☐ ID checks/badges (for students and teachers)

- [ ] ID checks/badges (visitors)
- [ ] Metal entrance turnstiles
- [ ] None of the above

Page Break

Are there any security countermeasures that you would feel **uncomfortable** with your school using? (Select all that apply)

- ☐ Security Camera
- ☐ Uniformed security or law enforcement officers
- ☐ Plain clothes (undercover) security or law enforcement officers
- ☐ Barriers to mitigate vehicles from plowing into crowds
- ☐ Walk-through metal detectors
- ☐ No firearms policy
- ☐ Bag inspection
- ☐ Clear bag policy
- ☐ Threat assessment programs
- ☐ Interior door locks
- ☐ Faculty/Staff emergency plan trainings/drills
- ☐ Student emergency plan trainings/drills
- ☐ Armed faculty/staff
- ☐ Anonymous suspicious activity reporting system
- ☐ Bulletproof glass
- ☐ ID checks/badges (for students and teachers)

☐ ID checks/badges (visitors)

☐ Metal entrance turnstiles

☐ None of the above

Page Break

Please indicate how much you agree (or disagree) with the following statements regarding security countermeasures and safety at your school.

	Strongly Disagree	Somewhat disagree	Neither agree nor disagree/ Not Applicable	Somewhat agree	Strongly agree
Security cameras increase my safety & the students safety	○	○	○	○	○
Uniformed security officers increase my safety & the students safety	○	○	○	○	○
Traffic barriers increase my safety & the students safety	○	○	○	○	○
Walk through metal detectors increase my safety & the students safety	○	○	○	○	○
No firearms policies increase my safety & the students safety	○	○	○	○	○
Bag inspections increase my safety & the students safety	○	○	○	○	○
Clear bag policies increase my safety & the students safety	○	○	○	○	○
Threat assessment programs increase my safety & the students safety	○	○	○	○	○

Interior door locks increase my safety & the students safety	○	○	○	○	○
Faculty emergency training/drills increase my safety & the students safety	○	○	○	○	○
Student emergency training/drills increase my safety & the students safety	○	○	○	○	○
Armed faculty/staff increase my safety & the students safety	○	○	○	○	○
Anonymous suspicious activity reporting system increase my safety & the students safety	○	○	○	○	○
Bulletproof glass increases my safety & the students safety	○	○	○	○	○
ID checks/badges increase my safety & the students safety	○	○	○	○	○
Metal entrance turnstiles increase my safety & the students safety	○	○	○	○	○

Please indicate how much you agree (or disagree) with the following statements regarding countermeasures and privacy at schools in the United States.

	Strongly Disagree	Somewhat disagree	Neither agree nor disagree / Not applicable	Somewhat agree	Strongly agree
Security cameras invade my privacy & the students privacy	○	○	○	○	○
Walk through metal detectors invade my privacy & the students privacy	○	○	○	○	○
Bag inspections invade my privacy & the students privacy	○	○	○	○	○
Clear bag policies invade my privacy & the students privacy	○	○	○	○	○

Page Break

Please select 'strongly agree' to show that you are paying attention to this question.

- Strongly agree
- Agree
- Neither agree nor disagree
- Disagree
- Strongly Disagree

What year were you born?

▼ 2004 ... 1921

What is the highest level of school you have completed or the highest degree you have received?

- Less than high school diploma
- High school graduate (high school diploma or equivalent GED)
- Some college but no degree
- Associate's degree
- Bachelor's degree
- Master's degree
- Professional degree (J.D., M.D.)
- Doctoral degree

What is your sex?

- ○ Male
- ○ Female
- ○ Other
- ○ Prefer not to answer

Choose one or more races that you consider yourself to be:

- ☐ White
- ☐ Black or African American
- ☐ American Indian or Alaska Native
- ☐ Asian
- ☐ Native Hawaiian or Pacific Islander
- ☐ Hispanic or Latino
- ☐ Other ______________________________

Please give your best estimate of your typical annual household annual gross income (including any government assistance and before taxes or other deductions) in the past few years?

- ○ Less than $10,000
- ○ $10,000 - $19,999
- ○ $20,000 - $29,999
- ○ $30,000 - $39,999
- ○ $40,000 - $49,999
- ○ $50,000 - $59,999
- ○ $60,000 - $69,999
- ○ $70,000 - $79,999
- ○ $80,000 - $89,999
- ○ $90,000 - $99,999
- ○ $100,000 - $149,999
- ○ $150,000 or more

---

*

What is the ZIP code of the residence where you have lived in the last 12 months?

____

Here is a 7-point scale on which the political views that people might hold are arranged from extremely liberal (left) to extremely conservative (right). Where would you place yourself on this scale?

Extremely Liberal			Moderate			Extremely Conservative
1	2	3	4	5	6	7

Political Ideology	

Page Break

Please provide us with your feedback on the survey. We are interested in the following issues: Did you notice any misspellings, grammatical errors, or formatting issues throughout the survey? If yes, please list them.   Did you find any questions unclear? If yes, what are they?   Is the flow of the survey logically presented?   Did we miss anything that you thought we ought to include?

____

Appendix B

Correlations of Predictors for Logistic Regression Predicting Feelings of Safety

Students

Predictor	1	2	3	4	5	6	7	8	9	10	11
1.Satisfied	-	-	-	-	-	-	-	-	-	-	-
2.Unwanted	-.28	-	-	-	-	-	-	-	-	-	-
3.Add	-.54	-.51	-	-	-	-	-	-	-	-	-
4.Comfortable + Effective	.16	.23	.11	-	-	-	-	-	-	-	-
5.Uncomfortable + Ineffective	-.13	.23	-.15	-.59	-	-	-	-	-	-	-
6.Uncomfortable + Effective	-.15	.03	.03	-.07	-.06	-	-	-	-	-	-
7. School Type	.00	.02	.01	-.09	.05	.05	-	-	-	-	-
8. Sex	-.29	.01	.22	.12	-.10	.11	-.02	-	-	-	-
9. Race	.08	-.05	-.05	-.04	.00	-.02	-.08	.00	-	-	-
10. Income	.02	.03	-.06	.04	-.02	.02	.09	.01	-.17	-	-
11. Political Ideology	.07	-.04	.01	.02	-.01	-.23	.04	-.31	.05	.01	-
12. Education	-.05	.04	.01	.00	.05	.08	.04	.13	-.15	.10	-.07

Parents

Predictor	1	2	3	4	5	6	7	8	9	10	11
1.Satisfied	-	-	-	-	-	-	-	-	-	-	-
2.Unwanted	.04	-	-	-	-	-	-	-	-	-	-
3.Add	-.52	.00	-	-	-	-	-	-	-	-	-
4.Comfortable + Effective	.27	.04	.20	-	-	-	-	-	-	-	-
5.Uncomfortable + Ineffective	-.25	-.08	-.15	-.56	-	-	-	-	-	-	-

6.Uncomfortable + Effective	.01	.00	.00	-.16	-.04	-	-	-	-	-	-
7. School Type	-.06	.00	-.10	-.10	.02	.01	-	-	-	-	-
8. Sex	.05	-.16	.11	.02	.03	-.08	-.03	-	-	-	-
9. Race	.11	.02	.00	.05	-.01	.014	.02	.09	-	-	-
10. Income	.03	.05	-.17	-.06	-.02	.11	.08	-.21	-.13	-	-
11. Political Ideology	.00	.02	.02	.09	-.12	.00	.09	-.18	-.10	-.02	-
12. Education	.02	-.04	-.17	-.20	.13	.06	.16	-.12	-.05	-.48	-.05

Staff

Predictor	1	2	3	4	5	6	7	8	9	10	11
1.Satisfied	-	-	-	-	-	-	-	-	-	-	-
2.Unwanted	-.39	-	-	-	-	-	-	-	-	-	-
3.Add	-.47	-.49	-	-	-	-	-	-	-	-	-
4.Comfortable + Effective	.26	-.47	.20	-	-	-	-	-	-	-	-
5.Uncomfortable + Ineffective	-.26	.40	-.13	-.59	-	-	-	-	-	-	-
6.Uncomfortable + Effective	-.14	.17	-.11	-.35	.09	-	-	-	-	-	-
7. School Type	-.17	.13	.02	-.11	.14	-.01	-	-	-	-	-
8. Sex	-.12	-.01	.12	-.01	.02	.02	.01	-	-	-	-
9. Race	.11	-.14	.00	.06	-.01	.02	.00	.01	-	-	-
10. Income	.20	.05	-.20	.02	-.01	-.08	-.10	-.15	-.02	-	-
11. Political Ideology	.10	-.15	.10	.20	-.23	-.27	-.09	-.03	-.02	-.02	-
12. Education	.07	.02	-.11	-.02	-.01	.07	-.08	-.08	-.11	.20	.01
